# Association between Human Urotensin II and Essential Hypertension—A 1:1 Matched Case-Control Study

**DOI:** 10.1371/journal.pone.0081764

**Published:** 2013-12-10

**Authors:** Hao Peng, Mingzhi Zhang, Xiaoqin Cai, Jennifer Olofindayo, Anna Tan, Yonghong Zhang

**Affiliations:** 1 Department of Epidemiology, School of Public Health, Medical College of Soochow University, Suzhou, China; 2 Department of Diagnostic Center, the Kunshan Affiliated Hospital of Nanjing University of Traditional Chinese Medicine, Suzhou, China; 3 Tulane School of Public Health and Tropical Medicine, Epidemiology Department, New Orleans, Louisiana, United States of America; 4 Tulane University, Public Health Department, New Orleans, Louisiana, United States of America; Shanghai Institute of Hypertension, China

## Abstract

**Objective:**

We aimed to evaluate the controversial association between human urotensin II and essential hypertension in untreated hypertensive cases and normotensive controls.

**Methods:**

197 newly diagnosed hypertensive patients and 197 age- and sex-matched normotensive controls were studied. Plasma urotensin II, nitric oxide metabolites, and other traditional biomarkers were examined.

**Results:**

Hypertensive patients had higher urotensin II [median (interquartile rang): 9.32 (7.86–11.52) ng/mL *vs* 8.52 (7.07–10.41) ng/mL] and lower nitric oxide metabolites [19.19 (2.55–38.48) µmol/L *vs* 23.83 (11.97–43.40) µmol/L] than normotensive controls. Urotensin II was positively correlated with systolic blood pressure (r = 0.169, *P*<0.001) and diastolic blood pressure (r = 0.113, *P* = 0.024) while negatively correlated with nitric oxide metabolites (r = −0.112, *P* = 0.027). In multivariate regression analysis, subjects in the highest quartile of urotensin II were more likely to have hypertension than those in the lowest quartile (OR, 2.58; 95% CI, 1.21–5.49). Sub-group analyses in 106 pairs of cases and controls with either both normal or both abnormal nitric oxide metabolites levels showed that the association between urotensin II levels and hypertension persisted (*P* value for trend = 0.039).

**Conclusion:**

Human urotensin II is markedly associated with essential hypertension, and the association is independent of nitric oxide metabolites. Our results indicated that urotensin II might be an independent risk factor for essential hypertension.

## Introduction

Hypertension is a leading cause of cardiovascular morbidity and mortality and highly prevalent worldwide [1], [2]. The etiology of hypertension is imperfectly understood. Recently, the association between human urotensin II (UII) and hypertension has been examined by some case-control studies [3], [4], [5], [6], [7], with somewhat mixed results. Increased plasma UII has been found in hypertensive patients compared to normotensive individuals [3], [5], [6]. In contrast, some studies reported that UII levels in hypertensive patients were equal to [7] or lower [4] than UII levels in normotensive controls.

UII is a vasoactive peptide initially isolated from the neurosecretory system of the Goby fish [8] and later from humans [9]. It is involved in the regulation of the cardiovascular system in humans [9]. UII stimulates vasoconstriction or vasodilation depending on the vascular bed [10], [11] and on the condition of endothelium [9], [12].This might explain why the association between UII and hypertension are inconsistently reported in previous population-based studies.

Some antihypertensive medications have been shown to improve endothelial function [13], although the impact of endothelial function on UII levels is not clear. To date, endothelial function and antihypertensive medication have not been controlled for in previous studies. To examine the actual relationship between UII and hypertension, we studied the association while controlling for nitric oxide metabolites (nitrite + nitrate = NO_x_) in newly diagnosed untreated essential hypertensive patients and age- and sex-matched normotensive controls.

## Materials and Methods

### Study participants

**Figure 1:**
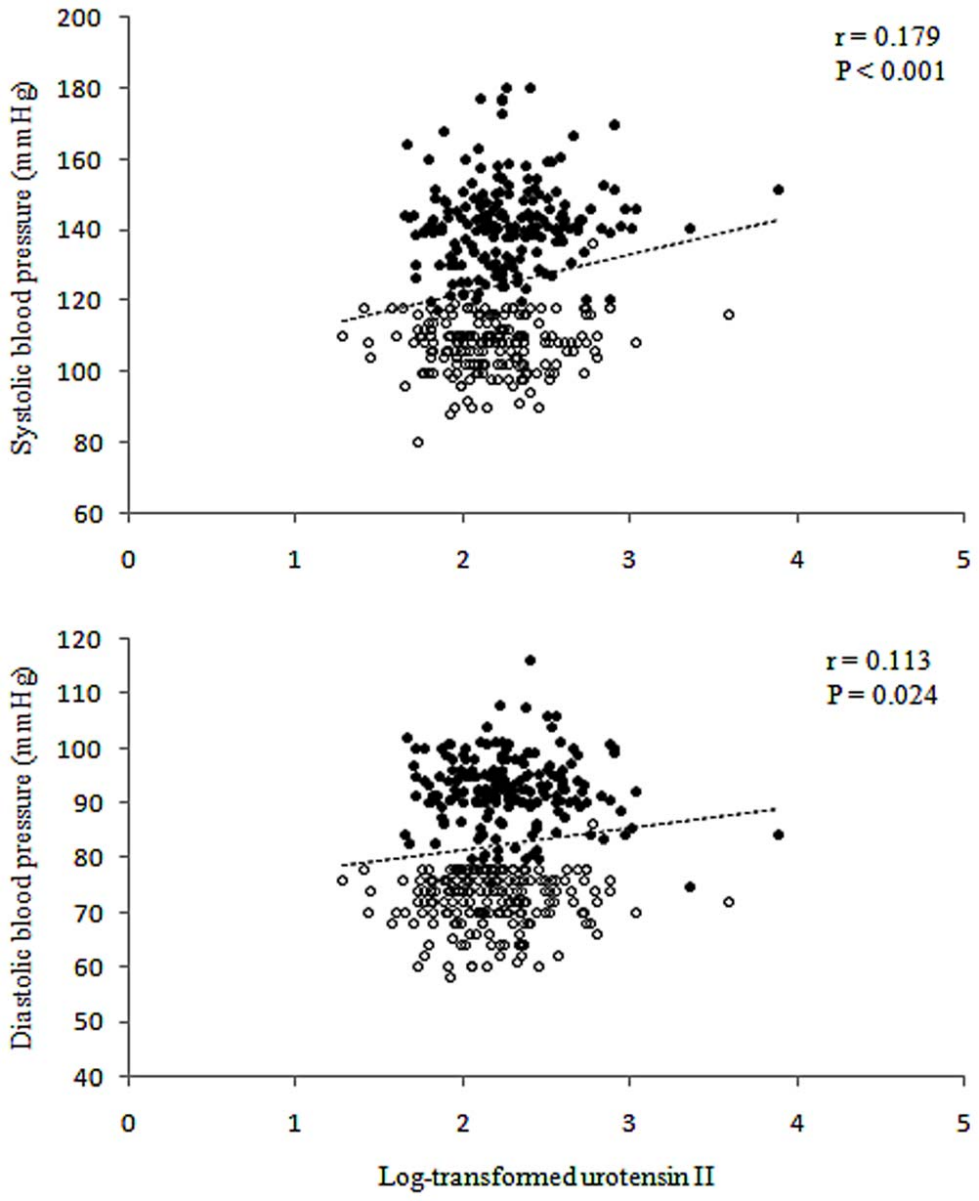
Correlation between plasma urotensin II and blood pressure. Closed circles represent hypertensive patients and open circles represent normotensive subjects.

A cross-sectional study was conducted in a population aged 30 years or more in Jinchang district of Suzhou city, China, from January to May of 2010. In this study, a total of 3061 participants were included after providing their written informed consent and blood samples. In the current study, hypertensive cases and normotensive controls were selected from the 3061 participants. Participants with one of the following were excluded: (1) coronary heart disease; (2) stroke; (3) tumors; (4) chronic kidney diseases (renal artery stenosis, coarctation, glomerulonephritis and pyelonephritis); (5) use of antihypertensive medication. Hypertension was defined as a systolic blood pressure (SBP) of 140 mmHg or more, or a diastolic blood pressure (DBP) of 90 mmHg or more and normotension was defined as SBP less than 120 mmHg and DBP less than 80 mmHg [14]. A total of 366 hypertensive and 515 normotensive subjects were eligible and further screened for cases and controls matched with age (±4 years) and sex by using SAS software. Finally, only 197 pairs were matched successfully and included in this study (Figure 1). This study was approved by the Soochow University Ethics Committee.

### Data collection

Data on demographic information, lifestyle risk factors, family history of hypertension, and personal medical history were obtained using a standard questionnaire written in Chinese and administered by trained staff. Cigarette smoking was defined as current smokers having smoked at least one cigarette per day for one year or more. Alcohol consumption was defined as consuming any type of alcoholic beverage at least once per week during the last three years. Body weight and height were measured by using a regularly calibrated stadiometer and balance-beam scale with participants wearing light clothing and no shoes. Body mass index (BMI) was calculated as weight in kilograms divided by the square of the height in meters. Waist circumference (WC) was measured at the level of one centimeter above the umbilicus. Three consecutive sitting blood pressure measurements (3 min between each) were taken by trained staff using a standard mercury sphygmomanometer according to a standard protocol [14] after the subjects had been resting for at least 30 minutes. The first and fifth Korotkoff sounds were recorded as SBP and DBP, respectively. The mean of the three readings was used in the analysis.

Blood samples were obtained in the morning by venipuncture after a requested overnight fasting (at least 8 hours) and sampled in EDTA tubes containing aprotinin and immediately spun for 15 minutes at 3,000 rpm. Plasma was frozen at −80°C until laboratory testing. Total cholesterol (TC), triglycerides (TG), high density lipoprotein cholesterol (HDL-C), low density lipoprotein cholesterol (LDL-C), and fasting plasma glucose (FPG) were analyzed enzymatically on a Hitachi 7020 automatic biochemical analyzer using commercial reagents (KANGXIANG MEDICAL APPliANCE, Shanghai, P.R China). NO_x_ was examined using a commercial kit (Nanjing Jiancheng Bioengineering Institute, Nanjing City, P.R China). Intra- and inter-assay coefficients of variation were less than 2% and 4%, respectively. In the current analysis, normal range of NO_x_ concentration in adults ranged from 11.5 to 76.4 µmol/L in males and 10.1 to 65.6 µmol/L in females [15].

Plasma UII measurements were performed in a FilterMax F5 Multi-Mode Microplate Reader (Molecular Devices, LLC. Sunnyvale, California, United States) using commercial enzyme-linked immunosorbent assay kits [UII (human)-EIA kit; Abcam plc. Cambridge, UK] according to the manufacturer's instructions. The kits have a sensitivity of 1 ng/mL and 0% cross-reactivity with urotensin I, urocortin or angiotensin II. All samples were processed in a duplicate assay. A standard curve was constructed from which the UII concentrations of unknown samples were determined. Intra- and inter-assay coefficients of variation were less than 4% and 9%, respectively.

### Statistical analysis

The statistical analysis was conducted using SAS statistical software (version 9.1, Cary, North Carolina). Baseline characteristics in hypertensive patients and normotensive controls were compared using a Student's t-test, Wilcoxon rank-sum test, or the chi-square test as appropriate. For lacking a normal distribution, the average level of UII and NO_x_ were compared between the two groups using a Wilcoxon rank-sum test. Log-transformation was then used to approximate the distribution of data to normal. A pearson correlation analysis was used to evaluate the correlations of blood pressure and log-transformed NO_x_ with log-transformed UII, respectively. Then, a stepwise multiple regression analysis was used to evaluate the linear association between UII and blood pressure with log-transformed UII as a dependent variable. Some underlying covariates such as age, sex, NO_x_, cigarette smoking, alcohol consumption, family history of hypertension, BMI, blood lipids, and FPG were independent variables. Univariate and multivariate conditional logistic regression models were performed to assess the association between levels of UII and hypertension. In the current analysis, all study subjects were categorized into quartiles of UII. Odds ratio (OR) and 95% confidence interval (CI) of hypertension were calculated for upper quartiles of UII with the lowest quartile as a reference. Trends in the ORs of hypertension across increasing UII categories were determined, modeling UII category as an ordinal variable. The potential covariates such as age, sex, cigarette smoking, alcohol consumption, family history of hypertension, BMI, TC, TG, FPG, and NO_x_ were included in the multivariate model. To determine whether the association between UII and hypertension was independent of NO_x_, the conditional logistic regression models were repeated in pairs of participants who had the same NO_x_ status, namely both subjects stood within reference values for NO_x_ or both exceeded the reference values. In the multivariate model, age, sex, cigarette smoking, alcohol consumption, family history of hypertension, BMI, TC, TG, and FPG were adjusted for. A two-tailed *P* value less than 0.05 was considered statistically significant.

## Results

### Baseline characteristics

We studied 197 hypertensive cases and 197 age- and sex-matched normotensive controls (Table 1). Hypertensive patients were more likely to have higher BMI, WC, TC, TG, LDL-C, FPG, and lower HDL-C than normotensive controls (all *P* values<0.05). There was no significant difference in average age, the proportion of family history of hypertension, cigarette smoking, and alcohol consumption between hypertensive and normotensive subjects (all *P* values>0.05). Moreover, UII concentration was significantly higher in hypertensive patients [median (interquartile range): 9.32 (7.86–11.52) ng/mL] than that in normotensive controls [8.52 (7.07–10.41) ng/mL] (*P* = 0.003). The average level of NO_x_ was significantly lower in hypertensive patients compared with that in normotensive controls (*P* = 0.027).

**Table 1:** Baseline characteristics of hypertensive cases and normotensive controls.

Variables	Normotensive (n = 197)	Hypertensive (n = 197)	*P* value
aAge, year	50.96 (8.33)	52.36 (9.01)	0.111
Male, n (%)	60 (30.46)	60 (30.46)	1.000
Cigarette smoking, n (%)	38 (19.29)	24 (12.18)	0.072
Alcohol consumption, n (%)	21 (10.66)	27 (13.71)	0.355
FHH, n (%)	50 (25.38)	62 (31.47)	0.180
aBody mass index, kg/m^2^	23.55 (3.96)	25.21 (2.64)	<0.001
aWaist circumference, cm	79.06 (7.91)	83.56 (7.60)	<0.001
bUrotensin II, ng/mL	8.52 (7.07–10.41)	9.32 (7.86–11.52)	0.003
bTotal cholesterol, mol/L	4.99 (4.55–5.66)	5.25 (4.68–5.82)	0.017
bTriglycerides, mol/L	0.95 (0.72–1.51)	1.35 (0.93–1.71)	<0.001
bLDL-cholesterol, mol/L	2.86 (2.42–3.36)	3.08 (2.66–3.59)	0.001
bHDL-cholesterol, mol/L	1.50 (1.26–1.77)	1.37 (1.17–1.62)	0.002
bFasting plasma glucose, mol/L	4.9 (4.6–5.3)	5.4 (5.0–5.9)	<0.001
bNitric oxide metabolites, umol/L	23.83 (11.97–43.40)	19.19 (2.55–38.48)	0.027

^a^ expressed as mean (SD).

^b^ expressed as median (interquartile range); FHH: family history of hypertension; LDL: low density lipoprotein; HDL: high density lipoprotein.

### Associations of UII with blood pressure and NO_x_

**Figure 2:**
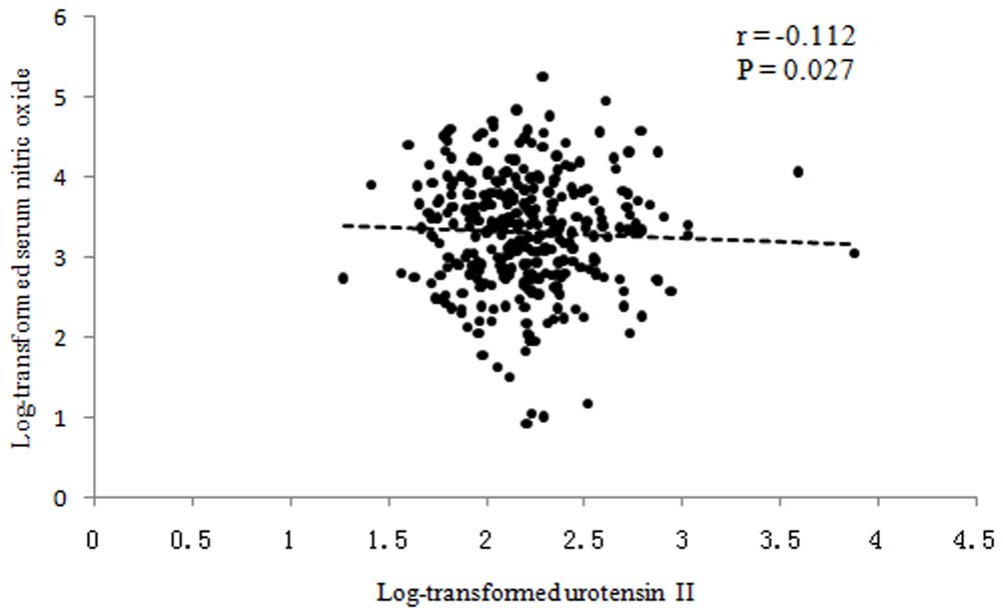
Correlation between plasma urotensin II and nitric oxide.

The pearson correlation analysis showed significant and positive associations of log-transformed UII with SBP (r = 0.169, *P*<0.001) and DBP (r = 0.113, *P* = 0.024), respectively (Figure 1), but on the other hand a significant and inverse correlation was found between log-transformed UII and NO_x_ (r = −0.112, *P* = 0.027) (Figure 2). In addition, the stepwise multiple regression analysis found that UII was significantly related to SBP (β = 0.002, *P*  = 0.003) and age (β  = 0.008, *P*<0.001) only (results not presented).

### Association between UII levels and hypertension

As shown in Table 2, univariate analysis showed a significant association between higher UII and hypertension (OR = 1.08, *P* = 0.015). After adjustment for potential covariates, risk of hypertension was still increased with UII although it was not significant (OR, 1.05; 95% CI, 0.98–1.12). When participants were categorized into quartiles of UII, ORs of hypertension for upper quartiles were calculated with the lowest quartile as a reference. In the univariate analysis, the OR of hypertension for subjects in the highest 25^th^ percentile of the distribution of UII had 2.85 times the OR of hypertension than individuals in the lowest 25^th^ percentile (*P* = 0.001). ORs of hypertension positively increased with UII level (*P* for trend = 0.001). In the multivariate analysis, association between UII levels and hypertension persisted (*P* for trend = 0.030). Risk of hypertension for individuals in the highest quartile of UII increased 158% compared with those in the lowest quartile (OR = 2.58, *P* = 0.014).

**Table 2:** Odds ratio and 95% confidence interval of hypertension associated with different plasma urotensin II levels.

Urotensin II (ng/mL)	OR (95%CI) of hypertension				
	Un-adjusted	*P* value		Adjusted	*P* value
Increase per unit	1.08(1.02–1.15)	0.015		1.05(0.98–1.12)	0.202
<7.30	1.00(reference)	–		1.00(reference)	–
7.31–9.00	1.49(0.85–2.61)	0.165		1.84(0.92–3.69)	0.084
9.01–10.90	1.75(0.96–3.20)	0.070		1.60(0.77–3.36)	0.211
≥10.91	2.85(1.52–5.34)	0.001		2.58(1.21–5.49)	0.014
*P* for trend	0.001			0.030	

Covariates for adjustment include age, family history of hypertension, cigarette smoking, alcohol consumption, body mass index, total cholesterol, triglycerides, fasting plasma glucose, and nitric oxide metabolites.

### Association between UII and hypertension after controlling for NO_x_

In addition to adjustment for NO_x_, sub-group analysis based on NO_x_ status was still needed to eliminate the potential influence of endothelial function on the association between UII and hypertension. We further excluded pairs of subjects who did not have matched NO_x_ levels. As a result, there were 78 pairs of subjects whose NO_x_ concentration were both within normal range and 28 pairs of subjects whose NO_x_ concentration both exceeded the reference range. Therefore, 106 pairs of cases and controls with matched NO_x_ status were finally used to verify whether the association between UII and hypertension was independent of NO_x_. As shown in Table 3, both before and after adjustment for age, cigarette smoking, alcohol consumption, family history of hypertension, BMI, TC, TG, and FPG, OR of hypertension significantly and positively increased with UII level (all *P* values for trend<0.05). After multivariate adjustment, individuals in the highest 25^th^ percentile of the distribution of UII were still more likely to have hypertension than those in the lowest 25^th^ percentile (OR = 3.31; *P* = 0.029).

**Table 3:** Odds ratio and 95% confidence interval of hypertension associated with different plasma urotensin II levels in nitric oxide level-matched pairs of subjects (106 pairs)^§^.

Urotensin II (ng/mL)	OR (95%CI) of hypertension				
	Un-adjusted	*P* value		Adjusted	*P* value
Increase per unit	1.11(1.02–1.21)	0.022		1.11(1.01–1.22)	0.041
< 7.30	1.00(reference)	–		1.00(reference)	–
7.31–9.00	1.30(0.62–2.70)	0.487		1.56(0.61–4.01)	0.358
9.01–10.90	1.76(0.80–3.88)	0.163		1.46(0.55–3.90)	0.449
≥10.91	3.08(1.33–7.15)	0.009		3.31(1.13–9.71)	0.029
*P* for trend	0.007			0.039	

Both subjects in 78 matched pairs were within normal reference values of nitric oxide metabolites (11.5–76.4 µmol/L for males and 10.1–65.6 µmol/L for females) and in 28 pairs both subjects exceeded the reference range.

Covariates for adjustment include age, family history of hypertension, cigarette smoking, alcohol consumption, body mass index, total cholesterol, triglycerides, and fasting plasma glucose.

## Discussion

This is a 1∶1 matched case-control study finding a positive and significant association between elevated plasma UII and essential hypertension. More importantly, this is the first to unearth that the association is independent of NO_x_. In our study, UII was elevated in hypertensive patients compared with normotensive controls. Conditional logistic regression analysis showed that subjects in the highest quartile of UII were more likely to have hypertension than those in the lowest quartile. In addition, pearson correlation analysis found that UII was positively correlated with both SBP and DBP but inversely correlated with NO_x_. As reported by Lim and colleagues [12], endothelial function might have an impact on the relationship between UII and hypertension. Hence, we further evaluated the association of UII with hypertension in NO_x_ level-matched hypertensive cases and normotensive controls. The results showed that the association between UII and hypertension remained significant. Moreover, UII was suggested to link the metabolic syndrome and other chronic diseases [16], [17]. In our study, in addition to exclusion of patients with chronic kidney disease and some other chronic diseases, confounders such as blood lipids, FPG and BMI were further controlled. Our results indicated that UII was significantly and positively associated with hypertension independently of NO_x_ and other confounders.

Indeed, association between UII and essential hypertension has been poorly studied in humans except for recently published studies with limited sample size [3], [4], [5], [6], [7]. Admittedly, in consistence with our study, some studies found that level of UII was increased in hypertensive patients compared to normotensive subjects [3], [5], [6]. Cheung and colleagues found that average UII level was elevated in 62 hypertensive patients compared with 62 age-matched normotensive controls; however, there were 37 patients receiving antihypertensive treatment [3]. It was unclear whether antihypertensive treatment affected the association between UII and hypertension, because the effect of antihypertensive drugs on urotensin levels has not been investigated. Interestingly, no significant difference in UII level was observed between 10 hypertensive patients receiving antihypertensive treatment and 10 normotensive controls [7]. We can see that antihypertensive drugs might have an influence on UII. Moreover, UII was found to be not only positively associated with SBP and DBP but also positively correlated with maximum intima-media thickness and carotid plaque score among elderly population [6], [18]. This indicated that vascular endothelial function may be involved in the association between UII and hypertension. No correlation between blood pressure and UII concentration was found among patients with kidney disease [4]. After dialysis, blood pressure reduced while plasma UII elevated in end-stage kidney disease patients [4], suggesting that UII caused vasodilation in humans with kidney disease. However, the mechanism involved in the vascular action of UII in kidney disease remained to be studied. The inconsistent reports about the association between UII and hypertension may be explained by different patient populations studied and the assays used, in fact, there is currently no consensus on a reliable method to measure plasma UII. In our study, all samples were processed in a duplicate assay with kits of the same batch, which minimized the experimental error. Despite the method, we also excluded individuals with co-existing diseases associated with elevated UII concentration [19], such as coronary heart disease and chronic kidney diseases. Unlike previous studies, a total of 394 subjects (197 hypertensive cases and 197 normotensive controls), the largest sample size to date, was included in our study, and all of the included cases were newly diagnosed hypertensive patients not receiving antihypertensive medication. In addition to adjustment for NO_x_, we further studied the association between UII and hypertension in NO_x_ level-matched pairs of subjects. We believe that the influence of antihypertensive drugs and endothelial function on UII was controlled for in our study.

Limitations of our study also merit consideration. First, this study was only a case-control design. Therefore, a causal relationship between UII and hypertension could not be established. Prospective cohort studies are warranted to further evaluate the causal relationship. Second, we only used levels of NO_x_ to evaluate endothelial function status in our study. Several other important biomarkers of endothelial dysfunction including Endothelin-1 and von Willebrand factor were not examined. Third, the controls were all normotensive individuals; prehypertensive participants were not included. Selection bias may not allow generalization to non-hypertensive participants, although significant correlations of UII with SBP and DBP were observed. Fourth, renal function, a confounder involved in the association between UII and hypertension [4], [19], was not evaluated in our study, although participants with chronic kidney diseases were excluded.

The underlying mechanism that explains why UII is elevated in hypertensive patients merits discussion. UII is a peptide first isolated in humans from subgroups of motor neurons in the spinal cord [20]. Its vascular actions are thought to be species and vascular bed specific [11]. Bohm et al. found that infusion of UII into the brachial artery caused vasoconstriction in vivo in healthy humans [21]. In contrast, UII was shown to be a strong vasodilator of human small muscular pulmonary arteries and human abdominal resistance arteries [22].

Interestingly, in patients with hypertension or diabetes, administration of UII caused a dose-dependent vasoconstrictor response in the forearm skin microcirculation [23], [24]. It is likely that enhanced contractility of UII is associated with endothelial dysfunction due to chronic diseases like hypertension or diabetes [25]. However, in our study, the association between UII and hypertension remained significant in NO_x_ level-matched pairs of subjects, indicating that the association between UII and hypertension is independent of endothelial function. Our results raised the possibility that UII may have an independent etiological role in hypertension and its complications. Human UII and its receptor might be candidate genes for hypertension. In light of our findings, antagonists to UII or its receptor might be antihypertensive agents with a novel mechanism action. Recently, a small clinical trial in only 54 hypertensive patients with type 2 diabetic nephropathy did not find significant effect of the urotensin receptor antagonist on blood pressure [26]. However, in the small clinical trial, all of the patients received renin-angiotensin-aldosterone system blockade, not allowing study of independent effects of urotensin receptor blockade. Therefore, an intensive clinical trial is needed to investigate the effect of human UII antagonists on hypertension treatment.

In conclusion, this study suggests that UII is significantly and positively associated with essential hypertension, and the association is independent of NO_x_. Prospective cohort studies and clinical trials are warranted to further evaluate the causal relationship.

## Supporting Information

Figure S1 **Selection of study subjects.**
(DOC)
