# Supplementary material for: Association between Human Urotensin II and Essential Hypertension—A 1:1 Matched Case-Control Study
Source: PLoS One. 2013 Dec 10;8(12):e81764. doi: 10.1371/journal.pone.0081764 (PMC3858253; doi:10.1371/journal.pone.0081764)
Supplement: Figure S1 — Selection of study subjects. (DOC) [file pone.0081764.s001.doc]

Supplementary Figure 1. Selection of study subjects

Total of 3061 subjects

1072 subjects were excluded

1989 eligible subjects

1108 prehypertensives

366 hypertensives

515 normotensives

197 pairs of hypertensive cases and age- and sex-matched controls with the same income level in the same community
